# Which target volume should be considered when irradiating the regional nodes in breast cancer? Results of a network-meta-analysis

**DOI:** 10.1186/s13014-019-1280-6

**Published:** 2019-06-11

**Authors:** Jan Haussmann, Wilfried Budach, Balint Tamaskovics, Edwin Bölke, Stefanie Corradini, Freddy-Joel Djiepmo-Njanang, Kai Kammers, Christiane Matuschek

**Affiliations:** 1 Department of Radiation Oncology, Heinrich Heine University, Dusseldorf, Germany; 2 Department of Radiation Oncology, LMU University of Munich, Munich, Germany; 3 Division of Biostatistics and Bioinformatics, Department of Oncology, The Sidney Kimmel Comprehensive Cancer Center at Johns Hopkins, The Johns Hopkins University School of Medicine, Baltimore, MD USA

**Keywords:** Breast cancer, Radiation therapy, Regional lymph nodes, Radiation volumes

## Abstract

**Purpose/objective(s):**

Radiation treatment to the regional nodes results in an improvement in survival in breast cancer according to a meta-analysis of randomized trials. However, different volumes were targeted in these studies: breast or chestwall only (WBI/CWI), inclusion of the medial supraclavicular region and axillary apex (MS + WBI/CWI) or additional inclusion of the internal mammary chain (IM + MS + WBI/CWI). The benefit of treating the medial supraclavicular region and axillary apex compared to tangential breast or chestwall irradiation only remains unclear.

**Materials/methods:**

A literature search was conducted identifying trials for adjuvant radiation volumes in nodal irradiation after breast surgery and axillary treatment. Events and effect sizes were extracted from the publications for the endpoints of overall survival (OS), breast cancer-specific survival (BCSS), disease-free survival (DFS), distant metastasis-free survival (DMFS) and loco-regional control (LRC). A network meta-analysis was performed using MetaXL V5.3 with the inverse variance heterogeneity model.

**Results:**

We found two randomized studies (*n* = 5836) comparing comprehensive nodal irradiation to sole breast treatment as well as one randomized (*n* = 1407) and one prospective cohort study (*n* = 3377) analysing the additional treatment of the internal mammary chain against sole local and supraclavicular and axillary apex radiation. Compared to WBI/CWI alone the treatment of IM + MS + WBI/CWI (HR = 0.88; CI:0.78-0.99; *p* = 0.036) results in improved OS unlike MS + WBI/CWI (HR = 0.99; CI:0.86-1.14; *p* = 0,89). These results are confirmed in BCSS: IM + MS + WBI/CWI (HR = 0.82; CI:0.72-0.92; *p* = 0.002) and MS + WBI/CWI (HR = 0.96; CI:0.79-1.18; p = 0.69). PFS is significantly improved with the treatment of MS + WBI/CWI (OR = 0.83; CI:0.71-0.97; p = 0.019). Both nodal treatment volumes improve LRC (MS + WBI/CWI OR = 0.74; CI:0.62-0.87; p = 0.004 and IM + MS + WBI/CWI OR = 0.60; CI:0.43-0.86; *p* < 0,001). Yet only the internal mammary nodes provide a benefit in DMFS (MS + WBI/CWI HR = 0.97; CI:0.81-1.16; p = 0.74 and IM + MS + WBI/CWI HR = 0.84; CI:0.75-0.94; p = 0.002).

**Conclusion:**

Expanding the radiation field to the axillary apex and supraclavicular nodes after axillary node dissection reduced loco-regional recurrences without improvement in overall and cancer-specific survival. A prolongation in survival due to regional nodal irradiation is achieved when the internal mammary chain is included. This derives from a reduction in distant metastasis.

## Introduction

Radiation therapy is a key component in the multidisciplinary approach of breast cancer treatment after breast conserving surgery and achieves equal oncologic results to mastectomy alone [1]. Over the last decades, surgical and radiation treatment to the lymphatic drainage of breast tumors have also been implemented as standard of care. Traditionally, axillary lymph node dissection was performed to determine the accurate tumor stage and eliminate nodal metastasis. Recently, the paradigm has shifted to do less extensive surgery, such as sentinel node biopsy alone. Regarding supraclavicular radiation therapy, anterior/posterior opposing fields were matched to the tangential fields of the breast to treat the axillary apex as well as the supraclavicular nodes to cover micro-metastatic spread into these regions. A second lymphatic drainage system is located parasternal along the internal mammary vessels coalescing with the axillary chain at the intersection with the subclavian vein. Extended radical mastectomies including a dissection of the internal mammary nodes (IMN) have been abandoned due to high complication rates and dubious oncological benefits [2, 3]. Radiotherapy (RT) was also used to cover this lymphatic drainage site and resulted in equivocal results with reports of a potential benefits [4, 5] as well as no advantage [2, 6] or even harmful effects [7]. However, a meta-analysis by the EBCTCG, based on individual patient data, identified the role of postmastectomy radiation (PMRT) including a comprehensive nodal irradiation, and reported significant benefits for local control, disease-free and overall survival. In fact, the vast majority of the 22 studies included radiation of the internal mammary chain [8].

Contemporarily breast cancer surgery is often performed using an organ preservation approach followed by adjuvant radiation therapy [1]. It has been a matter of debate whether nodal irradiation provides any benefit after axillary node dissection (AND) in the era of effective systemic therapies. Additionally, the regions of highest risk in the axilla might already be covered by standard tangential field irradiation, as whole breast radiation therapy applies substantial doses to the lower axilla and might provide equal control to dissection in clinically node negative but sentinel node positive patients [9, 10].

Moreover, inclusion of the internal mammary nodes proves to be a challenging task for radiation oncologists, because it subsequently leads to higher lung and heart doses, which increase the risks for late adverse events like ischemic heart events, secondary lung cancers or pulmonary fibrosis [11–14]. Furthermore, no consensus was reached on the optimal treatment application. Current techniques range from anterior electron fields to mixed electron and photon beam techniques, partial wide tangents, intensity-modulated RT techniques or protons [15].

Due to the aforementioned difficulties, nodal irradiation is often limited to the supraclavicular nodes and the axillary region at risk. This analysis was conducted to evaluate the additional benefit of supraclavicular and axillary apex radiation compared to whole breast or chest wall irradiation alone, as well as comprehensive nodal irradiation in clinical practice.

## Methods

A literature research according to PRISMA guidelines was performed using the MEDLINE as well as EMBASE and EBM review platforms [16]. Moreover, we screened the major meetings for published abstracts.

Search criteria were randomized or prospective observational trials reporting on regional nodal irradiation compared to no regional radiotherapy with a median follow-up of more than five years and trial publication after the year 2009. The study population had to consist of patients suffering from non-metastatic breast cancer treated with regional irradiation in least one trial arm. The exclusion criteria and time range were chosen to ensure a relatively homogeneous radiation technique, as well as systemic therapies mimicking current standard of care. We excluded patients undergoing surgical dissection of the internal mammary lymph nodes, preoperative radiation therapy and studies that used non-standard systemic therapies.

All available data were extracted as hazard ratios or event rates. Assessment of toxicities in the included trials was attempted. The definition of the analyzed endpoints was adopted from the published trials. If hazard ratios were not reported an attempt was made to calculate the hazard ratios and their corresponding 95% confidence intervals according to the method published by Parmar et al. [17]. If both effect measures were available, we elected to compare hazard ratios as they are regarded as most appropriate in analyzing time-to-event data. Visual analysis of publication bias by creating funnel plots was available but is not presented here due to the low number of included trials. Endpoints of the comparison included overall survival (OS), breast cancer-specific survival (BCSS), disease-free survival (DFS), distant metastasis-free survival (DMFS) and loco-regional control (LRC).

Data were analyzed using the Microsoft Excel plug-in MetaXl V5.3 and the included network meta-analysis function. Due to possible heterogeneity of the study populations the inverse variances of heterogeneity model (ivhet) by Doi et al. was chosen as the comparison method [18]. This method favors larger trials, uses a more conservative estimation of the confidence limits and produces lesser observed variances compared to the random effects model. Zero event correction was applied, where appropriated [19]. An intended analysis of heterogeneity was not feasible because not enough studies were available to form a closed loop. Subgroup analysis of matching endpoints and cohorts was intended. However, subgroups were only analyzed when two or more trials reported results in the specific subgroup.

Furthermore we performed a subgroup analysis according to the radiation volume of the EBCTCG individual patient meta-analysis on the effect of postmastectomy radiation [8]. We identified two studies that did not include the internal mammary region in the regional irradiation volumes. We extracted the numbers under risk and events from the different nodal disease subgroups from these two studies and compared them to the remaining trials in that specific subgroup. To avoid bias of analysis only subgroups with more than five patients per comparison were analyzed. Afterwards, we performed two comparisons (Comparison 1: chest wall irradiation + comprehensive nodal irradiation vs. no PMRT; Comparison 2: chest wall irradiation without IMN irradiation vs. no PMRT) using the same methods as described above. In the EBCTCG Analysis we included any first locoregional and any first recurrence in addition to overall survival as endpoints.

## Results

**Fig. 1:**
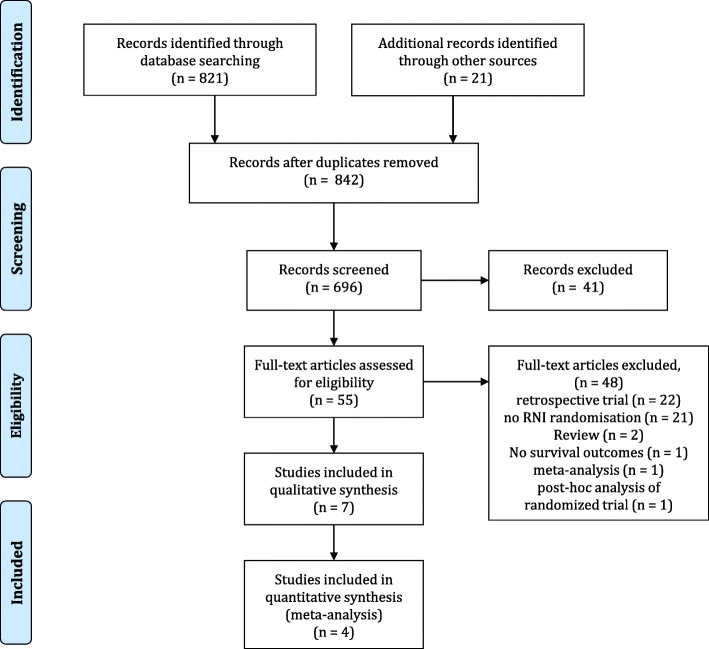
The PRISMA flowchart of the included trials

The literature search depicted in Fig. 1 identified four randomized or prospective trials matching the search criteria [20–23]. The EORTC trial was recently presented in an updated version, hence we used the available recent results in this analysis [20]. For non-updated endpoints the fully-published results were used [20]. Additionally, the EBCTCG meta-analysis was also considered to provide valuable information on the volumes used in PMRT compared to no adjuvant radiation therapy.

An overview of the included trials is presented in Table 1. Overall 10,620 patients with nodal positive or nodal negative with risk factors for lymphatic spread were randomized in the studies. The majority of included patients had pT1 or pT2 tumors with pN0 or pN1 disease. The surgical approach consisted of mastectomy (MTX) or breast-conservation surgery (BCS) and planned axillary lymph node dissection. The sentinel node approach only was used in fewer than 3% of the patients. Chemotherapy was regularly administered in the majority of trial participants. Median follow-up was above 8 years in all included trials. Table 2 shows an overview in which trials overall survival was analyzed between different subgroups.

**Table 1:** Overview of included trials. n.r. = not reported, FU = follow up, HR + = hormone receptor positive, N+/− = lymph node positive/negative, RN = regional nodes, IMN = internal mammary nodes, TV = target volume; ICS = intercostal space; BCS = breast conserving surgery; MTx = Mastectomy; AND = axillary node dissection

Trial	Synonym	Years trial	N total	med. Age	FU (y)	Post meno- pausal	HR+	CTx	N+	N-	T1	T2	Lateral Location	Surgery	Dose Breast	Dose RN	RT Boost	IMN TV	Main trial group
Hennequin 2013	French	1991–1997	1407	n.r.	8.6	n.r.	52%	61%	75%	25%	33%	52%	36%	MTx 100 AND 100%	50Gy	18 × 2.5 Gy =45Gy	none	ICS 1–5	N0 med/ central or N+
Thorsen 2016	DBCG- IMN	2003–2007	3377	56	8.9	60%	80%	53%	100%	0%	41%	52%	60%	BCS 35% MTx 65 AND 100%	24x2Gy =48Gy	24x2Gy =48Gy	13%	ICS 1–4	N+
Poortmans 2015/2018	EORTC 22922	1996–2004	4004	54	15.7	59%	78%	55%	56%	45%	60%	36%	n.r.	BCS 76,1% MTx 23,9% AND 100%	25x2Gy =50Gy	25x2Gy =50Gy	85%	ICS 1–3 (−4)	N0 med/ central or N+
Whelan 2015	Ma.20	2000–2007	1832	54	9.5	nr	75%	91%	90%	10%	52%	46%	62%	BCS 100 AND 96% SNB 4%	25x2Gy =50Gy	25 × 1.8–2 Gy =45-50Gy	33%	ICS 1–3	N0 (high risk) or N+

**Table 2:** Overview of subgroups analysed for overall survival by trial. a) Subgroup analysis based on (Poortmans et al. [20])

Subgroups	Thorsen	Hennequin	Poortmans^a^	Whelan
Primary Tumor Size				
-T1	**+**	**–**	**+**	**–**
-T2	**+**	**–**	**+**	**–**
-T3	**+**	**–**	**+**	**–**
Nodal Stage				
-N+	**+**	**+**	**+**	**+**
-N0	**–**	**+**	**+**	**+**
-N1	**+**	**–**	**+**	**+**
-N2+	**+**	**–**	**+**	**+**
Tumor Location				
-Medial / central	**+**	**+**	**–**	**+**
-Lateral	**+**	**+**	**–**	**+**
Hormonal Status				
-Premenopausal	**+**	**–**	**+**	**–**
-Postmenopausal	**+**	**–**	**+**	**–**
Type of Surgery				
-Mastectomy	**+**	**–**	**+**	**–**
-Breast conservation	**+**	**–**	**+**	**–**
Receipt of Chemotherapy	**–**	**+**	**+**	**–**

**Fig. 2:**
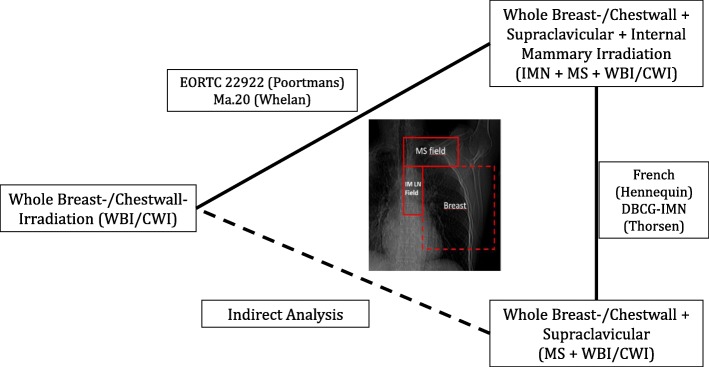
Overview of analyzed network according to target volume of regional irradiation

In Fig. 2 we present the resulting network for comparison. Two trials (EORTC 22922 and Ma.20) compared comprehensive regional nodal radiation (IMN + MS + WBI/CWI-RT) to whole breast−/chest wall-irradiation only (WBI/CWI-RT). The other two trials compared nodal radiotherapy with (IMN + MS + WBI/CWI-RT) and without (MS + WBI/CWI-RT) the parasternal lymph nodes (French and DBCG-IMN). Subsequently we were able to perform an indirect analysis of the comparison breast−/chest wall irradiation only (WBI/CWI-RT) vs. breast−/chest wall-irradiation + supraclavicular / axillary apex (MS + WBI/CWI-RT).

**Fig. 3:**
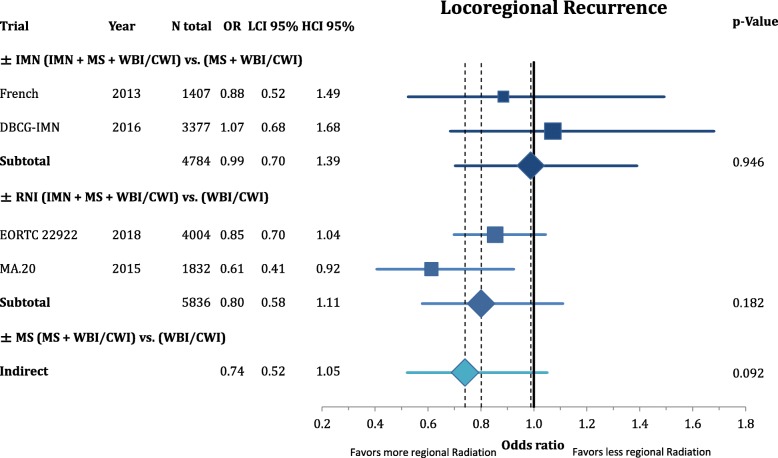
Forest plot of direct and indirect comparison of locoregional recurrence according to extend of regional radiation

**Fig. 4:**
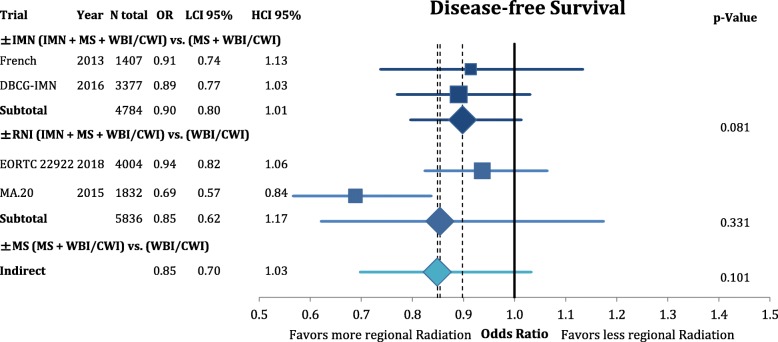
Forest plot of direct and indirect comparison of disease-free survival according to extend of regional radiation

**Fig. 5:**
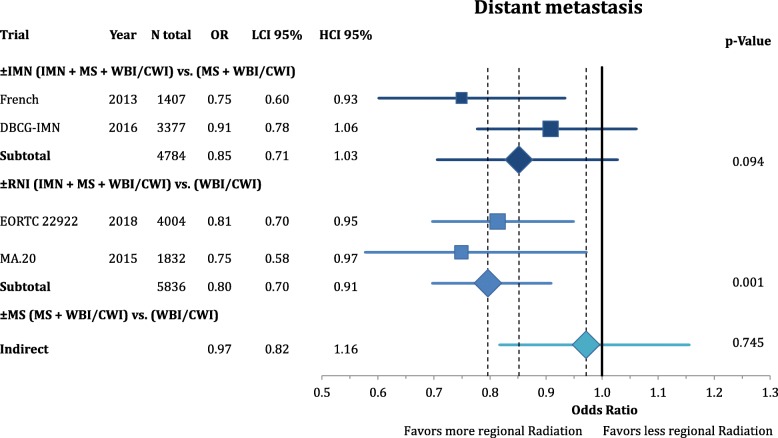
Forest plot of direct and indirect comparison of distant metastasis according to extend of regional radiation

**Fig. 6:**
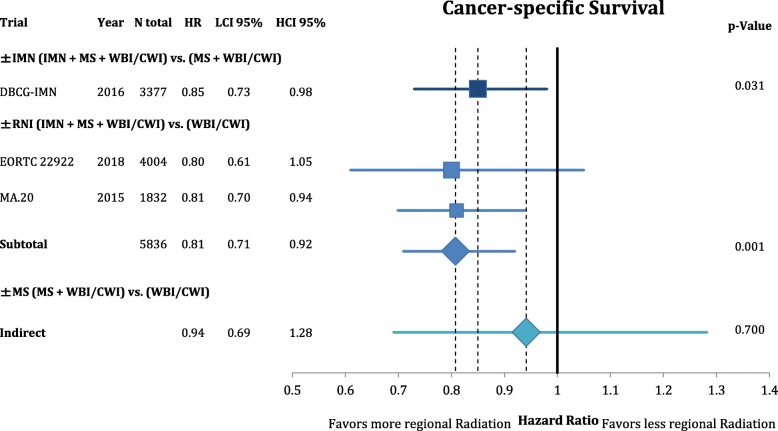
Forest plot of direct and indirect comparison of cancer-specific survival according to extend of regional radiation

**Fig. 7:**
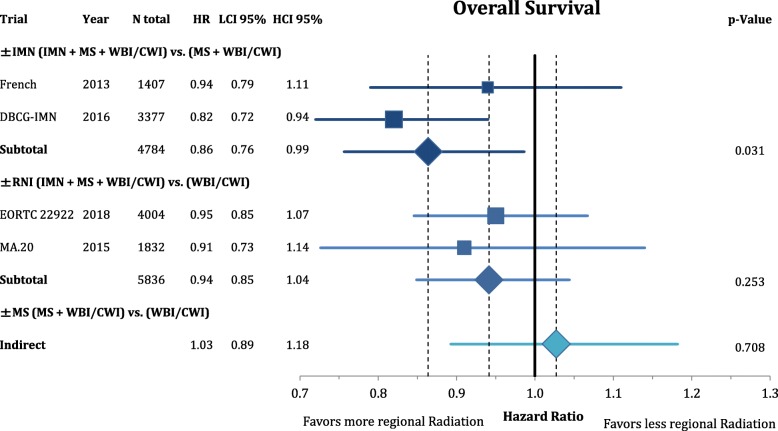
Forest plot of direct and indirect comparison of overall survival according to extend of regional radiation

According to Fig. 3, comprehensive RNI improved the rate of locoregional recurrence (OR = 0.80 CI95%: 0.68–1.11; *p* = 0.182). This effect was mainly based on the inclusion of the MS (OR = 0.74 CI95%: 0.62–1.05; *p* = 0.092) and not the IMN (OR = 0.99 CI95%: 0.70–1.39; *p* = 0.946) target volume. However, the endpoint of disease-free survival (Fig. 4) was not significantly improved by any components of the regional radiation **(+/− IMN**: OR = 0.90 CI95%: 0.80–1.01; *p* = 0.081; **+/− MS**: OR = 0.85 CI95%: 0.70–1.03; *p* = 0.101; **+/− RNI**: OR = 0.85 CI95%: 0.62–1.17; *p* = 0.331). This numeric improvement resulted from a significant reduction of distant metastasis from RNI (OR = 0.80 CI95%: 0.70–0.91; *p* = 0.001). IMN radiation showed a trend for an improvement in distant recurrence (OR = 0.85 CI95%: 0.71–1.03; *p* = 0.094), whereas MS-radiation had no impact (OR = 0.97 CI95%: 0.82–1.16; *p* = 0.745) (Fig. 5). As depicted in Fig. 6 this resulted in a significant improvement in breast-cancer specific survival in IMN-RT (HR = 0.85 CI95%: 0.73–0.98; *p* = 0.031) and RNI (HR = 0.81 CI95%: 0.71–0.92; p = 0.001). MS-RT had no significant effect (HR = 0.94 CI95%: 0.69–1.28; *p* = 0.700). Subsequently, overall survival was equally improved by IMN-RT (HR = 0.86 CI95%: 0.76–0.99; p = 0.031). After the inclusion of the 2018 update of the EORTC trial RNI did no longer significantly improve overall mortality (HR = 0.94 CI95%: 0.85–1.04; *p* = 0.253). Again MS-RT had no effect on survival (HR = 1.03 CI95%: 0.89–1.18; *p* = 0.708) (Fig. 7).

**Fig. 8:**
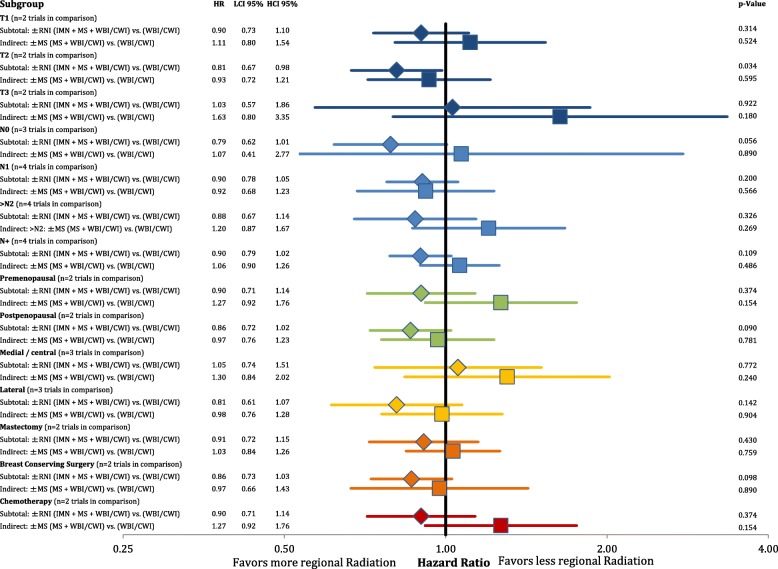
Forest plot of direct and indirect comparison of overall survival between different subgroups according to extend of regional irradiation. Direct comparison is depicted as diamonds, indirect comparison as squares

Subgroup analysis on overall mortality was feasible in a subset of trials (Table 2). Figure 8 shows the effect of comprehensive regional therapy compared to no regional RT as well as the indirect effect of MS + WBI/CWI-RT compared to WBI/CW-RT. We identified a significant improvement in overall survival by IMN + MS + WBI/CWI-RT in patients with T2 stage cancers. A statistical trend was seen in node negative, postmenopausal and patients treated with BCS. The relative effectiveness analysis revealed no subgroup that significantly profited in terms of overall mortality from MS + WBI/CWI-RT alone. Importantly, in all subgroups the estimated effect sizes were superior in patients treated with comprehensive nodal radiation therapy.

**Fig. 9:**
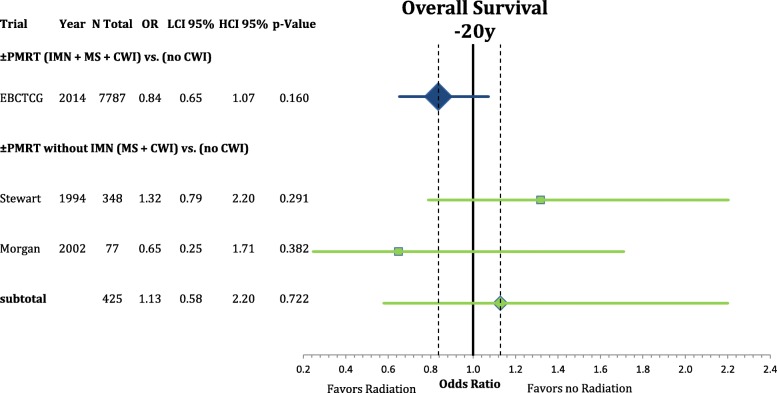
Forest plot of direct comparison of overall survival according to extend of regional radiation in the 2014 EBCTCG Meta-Analysis. Here, the comparisons are PMRT (IMN + MS + CWI) vs. no radiation and PMRT without IMN (MS + CWI) vs. no radiation

**Fig. 10:**
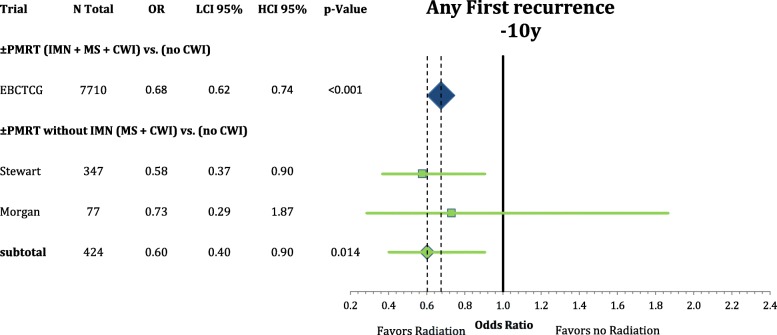
Forest plot of direct comparison of any first recurrence according to extend of regional radiation in the 2014 EBCTCG Meta-Analysis. Here, the comparisons are PMRT (IMN + MS + CWI) vs. no radiation and PMRT without IMN (MS + CWI) vs. no radiation

Furthermore we analyzed the effect of the sub-volumes in PMRT radiation compared to no PMRT in the individual patient meta-analysis by the EBCTCG published in 2014 [8]. We identified two trials that specifically did not include the internal mammary nodes in the postmastectomy radiation volumes and compared them to the included trials treating matching patient populations. Any first recurrence after 10 years and any death after 20 years results are depicted inn Figs. 9 and 10 comparing comprehensive PMRT and PMRT without IMN to no PMRT at all. PMRT with the inclusion of the IMN significantly improved the rate of any first recurrence after 10 years (OR = 0.68 CI95%: 0.62–0.74; *p* < 0.001) but did not improve the rate of any death after 20 years (OR = 0.84 CI95%: 0.65–1.07; *p* = 0.160). PMRT without the treatment of the IMN in the two included studies showed a significant improvement in the recurrence rate after ten years (OR = 0.60 CI95%: 0.40–0.90; *p* = 0.014). However, this did not translate into better survival (OR = 1.13 CI95%: 0.58–2.20; *p* = 0.722). As the analysis of locoregional recurrence showed equal results to any first recurrence it was not reported in detail.

**Fig. 11:**
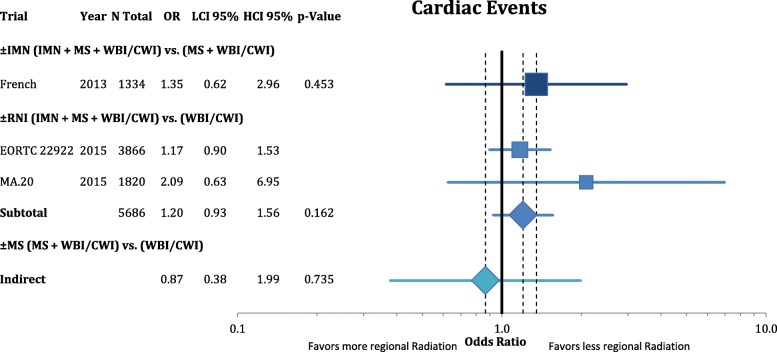
Forest plot of direct and indirect comparison of cardiac events according to extend of regional radiation. Cardiac events varied between studies and included acute myocardial infarction, ischemic heart disease, congestive heart failure, supraventricular arrhythmias and pericardial effusion

Figure 11 shows the analysis of cardiac events. We found no significant differences between the radiation volumes. However the point estimates imply that the non-significant increase in cardiac events derive mainly from the parasternal radiation.

## Discussion

Regional nodal irradiation in presumed intermediate risk breast cancer patients in stages I-III results in a small improvement in breast cancer specific and overall survival due to a reduction in distant metastasis [24]. This network meta-analysis supports the role of internal mammary radiation as the critical sub-volume to achieve these benefits. The supraclavicular irradiation appears to provide a benefit in locoregional control but does not, in contrast to the internal mammary nodal radiation, reduce the distant recurrence rate and subsequently mortality. This interpretation is supported by the additional investigation of the PMRT meta-analysis, showing a comparable reduction in any first recurrence with supraclavicular radiation. However, a trend towards improved mortality is only observed when the IMN are included in the radiation plan.

Despite the advantage in reducing metastases, the treatment of the internal mammary nodes is technically challenging and is accompanied by a significantly higher dose to the heart and lungs. Hence, the possible gain in survival has to be weighed against its harms, especially in patients with a history of smoking or other cardiovascular risk factors. These patients might not benefit from regional radiation at all, due to the higher absolute risks for secondary lung cancer or cardiac events [12, 13]. Our analysis demonstrates that, although not statistically significant, especially the increase in cardiac risks derive mainly from the parasternal radiation. RNI was reported to cause a small increase in pulmonary toxicity with higher event rates of pulmonary fibrosis and pneumonitis, as well as lymphedema [20, 22]. Additional treatment of the IMN did not resulted in a significant increase of Grade 3–4 adverse events [21]. A more thorough analysis of side effects separated by sub-volumes would be desirable but is currently not feasible, due to a lack of reported data.

The applied radiation techniques in the included trials ranged from 2D to early computer tomography-based approaches. However, modern techniques to reduce doses at organs at risk, like deep inspiration breath hold, field in field treatments or volumetric modulated arc therapy were not used [25]. The use of these techniques has been shown to further improve the therapeutic gain in RT. [26] Particularly the decreased benefit of regional radiation in the EORTC 22922 trial due to currently unexplained deaths raises some questions [27]. Efforts are currently being made to explain these unexpected events. Speculatively, late vascular side effects leading to cardiac disease (IMN fields) or cerebrovascular disease (supraclavicular fields) may have contributed to these observations [27–29].

It has been hypothesized that the benefit of any local treatment is dependent on the benefit resulting from systemic therapies [30]. The administered chemo- and hormone therapy in the included trials were heterogeneous. In the Ma.20 trial 90% of patients received adjuvant chemotherapy, mostly anthracycline-based, with about 25% receiving also taxanes [22]. In contrast, only around 55% of patients in the EORTC trial and in the Danish trial were treated with chemotherapy [20, 23]. In the French trial around 60% were treated with mainly anthracycline-based chemotherapy [21]. The effectiveness of regional irradiation in the Ma.20 trial, where the highest rate and closest to the current standard chemotherapy was offered, does not appear to differ substantially from the other trials. This is why we think that even modern systemic therapies do not mitigate the effectiveness of regional radiation.

Since the publication of the ACOSOG Z0011 trial, the routine use of axillary lymph node dissection (ALND) for the clinically node negative axilla has been declining [9]. However, the role of radiation therapy in this context is not well established [31]. The AMAROS and OTOASOR trials showed that radiation could replace axillary surgery and achieve equivalent effectiveness [10, 32]. With the routine omission of ALND, it is certainly possible that the RT treatment of the axilla and the supraclavicular region could regain more importance in the future.

What are possible explanations for the proposed differential oncologic effects of RT to the two components of the lymphatic chain? Obviously, the therapeutic approaches to both regions in the included studies were very different, since the axillary chain was dissected followed by irradiation and the internal mammary nodal chain was solely treated with radiation. The effect of MS radiation was investigated in large retrospective series demonstrating an impairment in DMFS and OS when supraclavicular recurrences occur during follow-up. However, on multivariate analysis RNI to the MS was not associated with an improvement in BCSS or OS, which reflects the results of the present analysis [33]. This deviation might be explained by alterations in the trial populations. The highest risk of supraclavicular involvement has been reported in patients with multiple axillary nodes, large nodal size, lymphovascular invasion, higher grading and extracapsular extension [33–35]. This high-risk population might be underrepresented in the present analysis, as less than 10% of the patients had pN2+ axillary staging.

Furthermore, since the publication of the ACOSOG Z0011 trial, it has been well established that a small tumor burden in the axilla can safely remain un-dissected and treated with systemic therapy [9]. Radiation therapy to the breast or chest wall might additionally contribute to this favorable outcome. It is possible that the two regions may be differently affected by these “incidental” treatments. Hormone- or chemotherapy might affect a postoperative region differently than a solely irradiated one. Moreover, the incidental radiation doses to the axillary levels I and II were reported to be larger than respectively to the IMNs [36–38].

The difference in locoregional control rates between MS- and IMN-RT fields could also be explained by the way recurrences are diagnosed in the clinical follow-up. As recurrences in both regions are usually subclinical and routine diagnostic imaging is often not included in the routine follow-up, the diagnosis of a regional recurrence is often delayed and accompanied by distant metastatic disease. Furthermore, regional relapses in the internal mammary nodes are very difficult to distinguish radiologically from mediastinal lymph nodes, leading to the diagnosis of metastatic disease. This influences the relative effects on locoregional recurrence and distant metastasis, but does not explain the impact on overall mortality.

The effectiveness of the IMN radiation is currently mainly explained by two hypotheses. IMN irradiation reduces the spread of micrometastases along this drainage site and subsequently lowers the risk for distant metastases. Furthermore, also an abscopal response, with a tumoricidal effect on non-target tumor cells, has also been postulated [24, 39]. One might expand this approach concluding that only IMN and not MS treatment can generate this effect, which explains our results. Currently the systemic effects of RNI and its clinical implications are insufficiently understood.

This network meta-analysis has several strengths and limitations we need to address. It includes high-quality trials with a considerable number of participants. Furthermore, the observed effects are relatively consistent among the subgroups, supporting the general conclusions of the analysis. One limitation is that we did not identify any trial addressing the effect of supraclavicular radiotherapy alone after surgical dissection. Consequently, the estimation of this comparison is only indirect and therefore hypothesis generating. Moreover, the inclusion of a prospective non-randomized trial increased the number of patients, but may add potential biases to the analysis [23]. However, restriction of the investigated endpoints to only randomized trials, showed no difference in outcome, providing support for the robustness of this analysis. The median follow-up between 8 and 15 years is relatively long but might still be inadequate to capture long-term side effects, like cardiac events or secondary carcinomas impacting mortality. Unfortunately, the analysis of adverse events was restricted to the cardiac event rates as the reporting was inconsistent in the included trials.

The comparison of multiple randomized trials in a network meta-analysis offers an intriguing option to investigate previously not directly compared treatment options. Like any meta-analysis the homogeneity of the included population, trial arms and investigated endpoints are central to a robust, meaningful analysis. Beyond the “classical” comparison of the pooled effect sizes, a network meta-analysis also allows a ranking of the groups by using the point estimates.

In view of the small benefit of nodal irradiation with an equally small but substantial risk of adverse events in an unselected patient population, it is of enormous importance to predict the benefit for different subgroups. Numerous attempts have succeeded in predicting which groups are at higher risk for local or distant relapse. However, to date there is no predictive test to estimate the benefit of radiotherapy. Subdivision by classical subtypes (hormone receptor, Her2, triple negative) produced varying results [40, 41]. Currently, tumor cells in the blood or bone marrow, as well as cellular markers scoring for intrinsic radiation sensitivity, are being studied to improve our understanding of the benefit of radiation [42–44].

Possible consequences and further topics of investigation might be a de-escalation of radiation volumes after an adequate ALND to the breast tissue and the internal mammary lymph nodes. To our knowledge, there have been no attempts to restrict the treatment to these volumes. An omission of MS-RT might decrease early and late toxicities, including esophagitis and radiation dermatitis, as well as hypothyroidism, pulmonary events and cardiac side effects [45]. Subgroups where this might be considered appropriate are patients with favorable biology as well as low axillary nodal burden.

This analysis should be viewed as hypothesis-generating for future investigations. Additionally, it could also provide a helpful guide for prioritization in the clinical practice, for example when dose constraints cannot be met or attempts to de-escalate radiation volumes are intended.

## Conclusion

Expanding the radiation field to the axillary apex and supraclavicular nodes after axillary node dissection reduced loco-regional recurrences without improvement in overall and cancer-specific survival. A prolongation in survival due to regional nodal irradiation is achieved when the internal mammary chain is included. This derives from a reduction in distant metastasis. 

## Abbreviations

ALND: Axillary lymph node dissection
AND: Axillary node dissection
BCSS: Breast cancer-specific survival
CI: Confident interval
CWI: Chest wall irradiation
DFS: Disease free survival
DMFS: Distant metastasis-free survival
EORTC: European Organisation for Research and Treatment of Cancer
FU: Follow up
HR: Hazard ratio
HR-: Hormone receptor negative
HR +: Hormone receptor positive
IMN: Internal mammaria node
Ivhet: Inverse variance of heterogeneity model
LCR: Loco-regional control
MTX: Mastectomy
N-: Lymph node negative
n.r: Not reported
N +: Lymph node positive
OS: Overall survival
PFS: Progression free survival
PMRT: Postmastectomy radiation
RT: Radiotherapy
WBI: Whole breast irradiation
